# Sensitivity of Bayesian Networks to Errors in Their Structure

**DOI:** 10.3390/e26110975

**Published:** 2024-11-14

**Authors:** Agnieszka Onisko, Marek J. Druzdzel

**Affiliations:** Faculty of Computer Science, Białystok University of Technology, Wiejska 45A, 15-351 Białystok, Poland; marek@bayesfusion.com

**Keywords:** Bayesian networks, graphical structure, sensitivity, medical diagnosis, accuracy

## Abstract

There is a widespread belief in the Bayesian network (BN) community that while the overall accuracy of the results of BN inference is not sensitive to the precision of parameters, it is sensitive to the structure. We report on the results of a study focusing on the parameters in a companion paper, while this paper focuses on the BN graphical structure. We present the results of several experiments in which we test the impact of errors in the BN structure on its accuracy in the context of medical diagnostic models. We study the deterioration in model accuracy under structural changes that systematically modify the original gold standard model, notably the node and edge removal and edge reversal. Our results confirm the popular belief that the BN structure is important, and we show that structural errors may lead to a serious deterioration in the diagnostic accuracy. At the same time, most BN models are forgiving to single errors. In light of these results and the results of the companion paper, we recommend that knowledge engineers focus their efforts on obtaining a correct model structure and worry less about the overall precision of parameters.

## 1. Introduction

Decision-analytic methods provide a coherent framework for modeling and solving decision problems in intelligent systems [1,2]. A popular modeling tool for complex uncertain domains is a Bayesian network (BN) [3], a directed acyclic graph that represents the structure of a domain that, jointly with numerical parameters, represents the joint probability distribution over its variables. BNs are grounded in probability theory and are theoretically sound while being intuitive at the same time. They are widely used in those artificial intelligence (AI) problems that require theoretical soundness and reliability. There exist algorithms for inference in BNs that derive the posterior probability distribution over variables of interest given a set of observations of other variables. Because these algorithms are mathematically correct, they essentially solve the underlying model; hence, the ultimate quality of the results depends directly on the quality of this model.

There is a popular belief among BN practitioners that, while the precision of parameters is not too important, it is crucial to have the structure right. While the question of BN sensitivity to noise in parameters has received considerable attention (e.g., [4,5,6]), to our knowledge, the sensitivity of BNs to errors in their structure has not been systematically studied. Several years ago [7], we presented the results of a study in which we performed systematic changes to the structure of several medical diagnostic BN models and measured how these changes affect their diagnostic accuracy. While our work has a handful of citations that refer to our results, there seems to be no follow up on the study itself.

This paper aims at filling this gap and offers a comprehensive extension of our prior work. We have amended our original experimental setup, extended the set of models tested, conducted additional experiments, and changed the quality measure, observing somewhat different results that are more in line with the popular belief in the BN community. In our experiments, we systematically introduced structural errors into a collection of medical diagnostic BN models and tested the diagnostic accuracy of the resulting models. Similar to both Pradhan et al. [4,6] and our earlier work, we assumed that the original models and their accuracy are a gold standard.

The main result of our analysis is that the structural errors in BNs may lead to a serious deterioration in model accuracy, especially if the errors concern important model elements. Still, there is a small region of, typically, a handful of errors that have minimal effect on the accuracy. While building BN models is the bottleneck in a decision–theoretic approach to AI, the structure seems to be much easier to obtain than numerical parameters [8]. Our results lead to some cautious optimism as it is easier to enhance something that is not too hard to start with.

The remainder of this paper is structured as follows. Section 2 provides a brief overview of the data sets (Section 2.1) and models (Section 2.2) used in our experiments. Section 2.3 outlines the architecture of our experiments, and Section 2.4–Section 2.6 describe our experiments and their results. Finally, Section 3 discusses these results in light of previous work, outlines the possible weaknesses of our study, and provides points of direction for further work.

## 2. Materials and Methods

For the purpose of our experiments, similar to Pradhan et al. [4,6] and our earlier work on the influence of the noise and precision of BN parameters on their accuracy [5,9], we started with existing BN models and assumed that they were perfect. Effectively, the diagnostic accuracy of the initial models was the best achievable by assumption. Of course, in reality, any model and its accuracy can be typically improved upon but this, we believe, does not undermine our experiments and their results as the initial models and their accuracy merely provide starting points and a comparison benchmark. In each of our experiments, we studied how this baseline accuracy degrades as a function of the number of structural errors.

There are several ways of measuring the diagnostic accuracy of a model. A simple measure that has been applied by Pradhan et al. [4,6] is to monitor the average posterior probability of the true diagnosis. We have shown in our companion paper that this measure does not reflect the accuracy of a diagnostic system well, and we proposed to use the percentage of correct diagnoses on real patient cases (ACC). This, as we will show in this paper, is a simplification that does not work too well in the case of structural changes. As an improvement on this measure, we propose to use the measures originating from the theory of information, notably the ROC (receiver operating characteristic) curves, for individual diagnoses. ROC curves look at individual diagnoses and show the relationship between sensitivity and specificity. A less precise but insightful measure that summarizes the overall quality of a model is the AUC, Area Under the (ROC) Curve, measure. Because the AUC is a real number between 0 and 1, it makes the comparisons of different models easy. ROC and AUC become somewhat complicated in the case of models focusing on multiple disorders—there is no single measure of accuracy but rather a measure of accuracy for every single disorder. Because our work is probing the space of possible errors and their impact on the overall model accuracy, we chose to focus on the AUC measure for the *a priori* most likely diagnosis, i.e., the diagnostic class with the highest prevalence. The reason for this choice is that the most prevalent class has the strongest representation in the data and, hence, gives the statistically most reliable results.

### 2.1. Medical Data Sets

For the purpose of our experiments, we needed models that come with real sets of patient cases. The task of finding such models is close to impossible. However, BNs can be learned from the data (both their structures and their numerical parameters), and most BN software (including GeNIe, ver. 4.1 and its API SMILE, ver. 2.2.5, which we used in our experiments) offer learning algorithms. Having access to a real medical data set thus allows us the automatic and almost instantaneous building of an associated, admittedly imperfect, model. To obtain a collection of models for our experiments, we searched through the public Machine Learning Repository at the University of California Irvine [10] for real medical data sets that are suitable for modeling diagnostic problems. We subsequently automatically built BN models using the standard BN learning algorithms available in GeNIe.

We identified eight data sets in the repository that were (1) medical data sets; (2) contained at least 100 records; (3) contained a single class variable (this variable represents the diagnosis, which is critical for building a diagnostic model); (4) did not have too many continuous variables (each of these variables had to be discretized and we wanted to minimize the possible spurious dependencies stemming from discretization); and (5) had 5% or fewer missing values as standard BN structure learning algorithms cannot handle them. We replaced all of the missing values (in five of the eight data sets) with states representing the absence of a symptom or “normal” value, which we found in our earlier work [11] to lead to the highest diagnostic accuracy in medical diagnostic systems. Table 1 summarizes the most important properties of these eight data sets from the point of view of model building.

Most of these data sets were rather small and contained small numbers of variables. While they were far from being ideal, their most important property was that they were real, i.e., they described real patient cases, along with their inherent noise and diagnostic difficulty.

### 2.2. Bayesian Network Models

**Figure 1:**
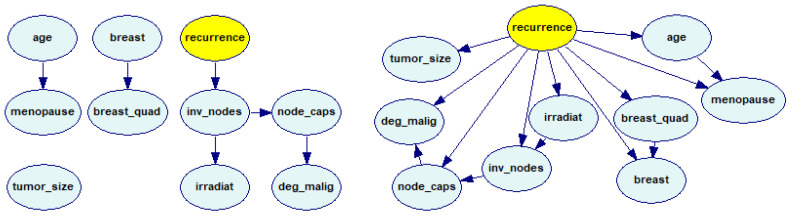
The BN models learned from the *Breast Cancer* data set: using the BSA algorithm (**left**) and using the ANB algorithm (**right**).

We used the selected data sets to learn the structures and the parameters of the medical models for our experiments. We constructed these models from the data sets listed in Table 1. We used the Bayesian Search (BSA) [19] structure learning algorithm implemented in GeNIe. In one case (the *Breast Cancer* data set), the BSA algorithm resulted in a model that consisted of four disconnected submodels (Figure 1, left). Its accuracy was also rather poor (please note that the class node, *recurrence*, was directly connected to only one node, *inv_nodes*). Because this is uncommon (the variables disconnected from the class variable are probabilistically unrelated to it and, hence, offer no information about classes), we decided, in this case, to use the ANB (Augmented Naive Bayes) algorithm instead (Figure 1, right). The ANB algorithm learns the model structure only partially, starting from a Naive Bayes structure and adding connections between the features to account for their conditional dependence given the class node.

Table 2 lists the models created for the purpose of our experiments. The model characteristics reported in the table give an idea of the complexity of the networks in terms of the number of nodes, the average number of states of these nodes, the average in-degree (i.e., the number of parents of a node, where the in-degree gives an idea of the connectivity of the network), the number of edges, and the number of numerical parameters. The networks and the data sets from which they were derived offered a reasonable test environment for our experiments.

### 2.3. Experimental Design

In our experiments, we focused on systematically introducing errors into the structure of the models. There were three possible structural errors: (1) the omission of a node, (2) the omission of an edge between two nodes, and (3) having a wrong edge orientation. In each case, we started with the gold standard structure (one of the models listed in Table 2), systematically introduced errors, and recorded the degradation in model accuracy.

Because a BN is a representation of the joint probability distribution, it contains all the information that is needed to derive measures such as the strength of an individual edge and probabilistic value of information (VOI) based on cross-entropy. In our experiments, we simulated three orderings over structural errors: (a) from the most to the least serious, (b) in a random order, and (c) from the least to the most serious. The meaning of the most and the least serious was different for nodes and edges—we will discuss the details of the orderings in the context of individual groups of experiments.

### 2.4. Node Removal

The main structural error that one can encounter in practice is omitted variables. We simulated this error by successively removing nodes of the gold standard networks and recording the effect that this had on the model accuracy.

It is possible to introduce ordering among removed nodes that is based on cross-entropy between the class variable (or variables) and each of the remaining nodes. Entropy H(Pr(x)), where Pr(x) is a probability distribution over a discrete variable *x* consisting of *n* states $\left(x_{1},x_{2},\ldots ,x_{n}\right)$, is defined as follows:

(1) $$H\left(\mathrm{Pr}\left(x\right)\right)=-\sum_{i=1}^{n}\mathrm{Pr}\left(x_{i}\right)\;\log\left(\mathrm{Pr}\left(x_{i}\right)\right).$$

Entropy reaches zero when all the probabilities are zeros and ones, which happens when there is no uncertainty, and it reaches its maximum when all probabilities are the same (i.e., for uniform distributions). It is common to interpret entropy as a measure of knowledge: the lower the entropy, the more we know; the higher the entropy, the more noise and the less knowledge known about the true state of the world.

The cross-entropy between two variables *x* and *y* is an information–theoretic measure that expresses the expected change in the entropy of the probability distribution over *x* after *y* has been observed. When both *x* and *y* are discrete variables with *n* and *m* states, respectively, cross-entropy H(Pr(x),Pr(y)) can be expressed as follows:

(2) $$H\left(\mathrm{Pr}\left(x\right),\mathrm{Pr}\left(y\right)\right)=H\left(\mathrm{Pr}\left(x\right)\right)-H\left(\mathrm{Pr}\left(x\right)|y\right)=H\left(\mathrm{Pr}\left(x\right)\right)-\sum_{i=1}^{m}\mathrm{Pr}\left(y_{i}\right)\;H\left(\mathrm{Pr}\left(x|y_{i}\right)\right).$$

Please note that we do not know *a priori* what the observation of *y* is going to be, and this is expressed by taking the expected value over the probabilities of all possible states of *y*. Cross-entropy, as expressed by Equation (2), will take positive values when the observation of *y* leads to a decrease in the entropy (i.e., offers more information about the state of *x*); otherwise, it takes on negative values.

When applied to the class variable *c*, the cross-entropy between *c* and any other model variable *y* expresses a probabilistic measure of the value of information (VOI) originating from *y*. It takes into account both the amount of information flowing from observing individual states and the probabilities of observing these states. The higher the value of cross-entropy, the higher the expected contribution of observing that variable to the probability distribution over *c*. Cross-entropy is a dynamic measure. Once we remove a node and retrain the new structure on the original data set, the order of the remaining nodes can change.

In the experiment, we simulated three orderings over node removal: (a) from the node with the lowest to the node with the highest cross-entropy, (b) random, and (c) from the node with the highest to the node with the lowest cross-entropy. It can be expected that removing nodes that are important (i.e., those that have the highest cross-entropy) will lead to a more rapid deterioration in diagnostic accuracy.

**Figure 2:**
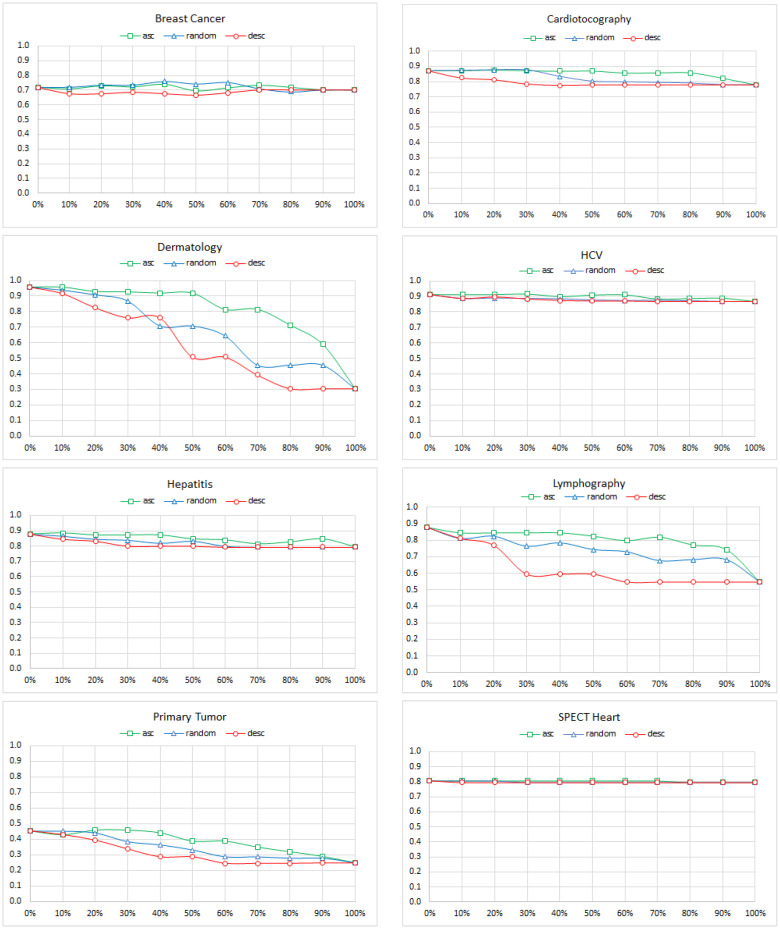
The ACC of the Breast Cancer, Cardiotocography, Dermatology, HCV, Hepatitis, Lymphography, Primary Tumor, and SPECT Heart models as a function of the percentage of nodes removed. The colors indicate the ascending, random, and descending order of the cross-entropy between the class node and the removed nodes.

As the first measure of diagnostic accuracy, we used the percentage of records for which the model guessed the correct class. The results (Figure 2) were quite surprising: it looked like, for a majority of the models, the diagnostic accuracy was not affected by omitted variables. This prompted us to analyze the results further and realize that our models used the posterior probabilities of various disorders as the diagnostic decision criterion. This led to always picking the most likely disorder, and this was seriously impacted by the prior probability (prevalence) of the disorder. When, for example, all of the nodes except for the class node had been removed (the extreme right in the plots), the model will essentially mimic the prevalence of the disease and will always choose the most prevalent disease as the correct class. This will result in an accuracy that equals to the probability of the most prevalent class; yet, it is a worthless model that offers 100% sensitivity but 0% specificity. Consider a model that aims at detecting a rare form of cancer with a prevalence of 0.000001 (one in a million). A model that predicts the *absent* state for all records will have an accuracy of 99.9999% but will fail to detect a single case of cancer and will be worthless. It can be shown easily that, under this measure, the model accuracy will never fall below the probability of the most prevalent class. The prevalence of various diseases in our models is quite asymmetric, as shown in Table 3 (the prevalence of the most probable class is in bold).

It is worth noting that the three models that had multiple classes with a more balanced prevalence of each class (Dermatology, Lymphography, and Primary Tumor) did show a clear deterioration of accuracy. While we used several of these same models in our experiments to test the impact of noise on the diagnostic accuracy, the problem was not critical there as the noise also impacted prevalence. Effectively, any disease could become a winner, and accuracy was the correct measure to use.

In our experiments, the node and edge removal and the edge reversal were followed by training the new structure with the original data set, which ensures that the joint probability distribution over the variables in the mutilated model did not depart from the original distribution. We can see in, for example, the results for the Breast Cancer model that the accuracy remained constant throughout the mutilation of the model. This tells us that we need a more insightful measure of model quality that looks at how the model manages with recognizing a disease. The most comprehensive measure of model quality is the ROC curve, which plots, for a given disease, its true positive rate (sensitivity) as a function of the false positive rate (its complement of specificity). The ROC curve always starts at point (0,0) and ends at point (1,1). For a perfectly accurate model, it goes through point (0,1), i.e., the point of 100% sensitivity and 100% specificity. Each point on the curve corresponds to a combination of sensitivity and specificity. It is possible to tune the decision-making process in the classifier to precisely achieve that combination. Thus, the ROC curve concisely expresses the model quality.

As we mutilated the original structures, we obtained a series of ROC plots, the comparison of which was daunting. Luckily, there is a closely related measure called AUC (Area Under the ROC Curve) that summarizes an ROC curve by a real number between 0 and 1. A perfect model has an AUC of 1.0 (please recall that the ROC curve goes in that case through points (0,0), (0,1), and (1,1)), and a worthless model has an AUC close to 0.5 (the ROC curve is close to the diagonal). One problem with ROC curves is that they are drawn for binary classes. They can still be used when we counterpose a selected class (a disease) against all other possible classes (diseases). We decided to study and plot the ROC curves for the most prevalent class in the model.

**Figure 3:**
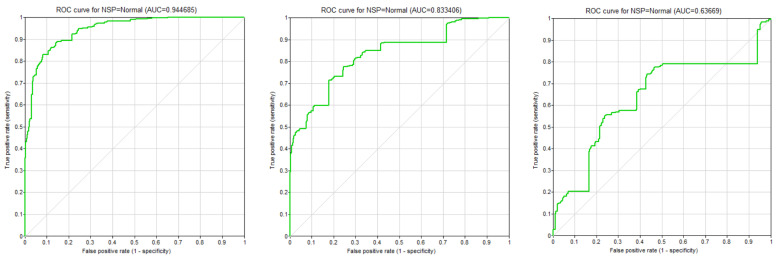
The ROC curves for the Cardiotocography model when 0%, 20%, and 40% of the nodes have been removed in a descending order.

Consider the series of ROC curves in Figure 3 for the Cardiotocography model, where class *NSP* = *Normal*, and for the gold standard model and models where 20% and 40% of the most important nodes have been removed. Please note that with more nodes removed, the ROC curve rapidly approaches the diagonal, indicating a close-to-worthless model. We see the AUC measure changed from the original 0.944685 through 0.833406 to 0.636690. Both, the shapes of the ROC curves and the AUC measures indicated a deterioration in the ability of this model to detect the *Normal* state of the *NSP* class node.

**Figure 4:**
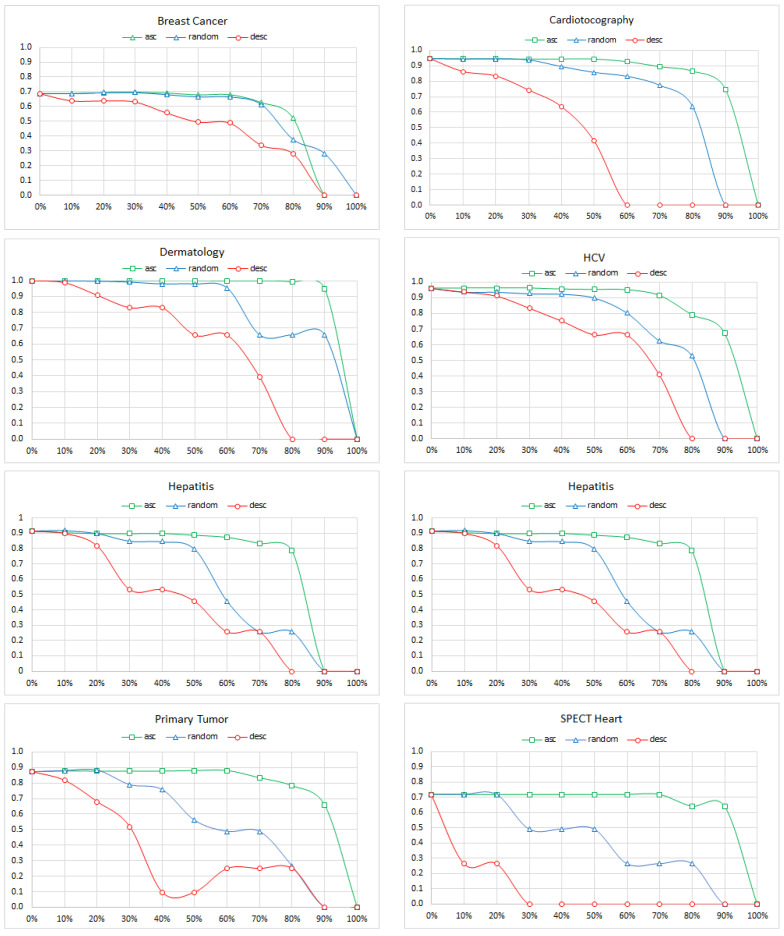
The AUC of the Breast Cancer, Cardiotocography, Dermatology, HCV, Hepatitis, Lymphography, Primary Tumor, and SPECT Heart models as a function of the percentage of nodes removed. The colors indicate the ascending, random, and descending order of the cross-entropy between the class node and the removed nodes.

We repeated our experiment with node removal, focusing this time on the AUC measure, which was calculated for the most prevalent class (its prevalence is marked bold in Table 3) after each node removal. Figure 4 shows the results. We can see from the figure that removing a handful of nodes had typically no immediate effect on the accuracy of the models. There was a small margin in our experiments where roughly 10–20% of the nodes had little effect on accuracy, even if they were important. Yet, for three of the models (Cardiotocography, Primary Tumor, and SPECT Heart), the effect of omitting important variables (i.e., those with the highest cross-entropy) led to an immediate deterioration in accuracy. The accuracy of all the models significantly decreased as more variables were omitted, especially when they were the most important variables. Accuracy was not affected too much when less important or randomly selected nodes were missing.

### 2.5. Edge Removal

Our next experimental manipulation of the BN structure involved edge removals. We mentioned earlier that the measurements of importance were different for nodes and edges. In the former case, we used cross-entropy. In the case of edges, we used a measure of arc strength. There were several measures of arc strength that were comprehensively reviewed in an M.Sc. thesis by Joost Koiter [20]. In our experiments, we used a measure of the arc strength proposed in Koiter’s thesis based on the differences between the posterior marginal probability distributions over the child node as the parent node changed. We chose the variant in which the differences were calculated in Euclidean space. Koiter’s measure was well grounded in the theory and we used it in the past in practical applications, as well as in our experimental work [7].

**Figure 5:**
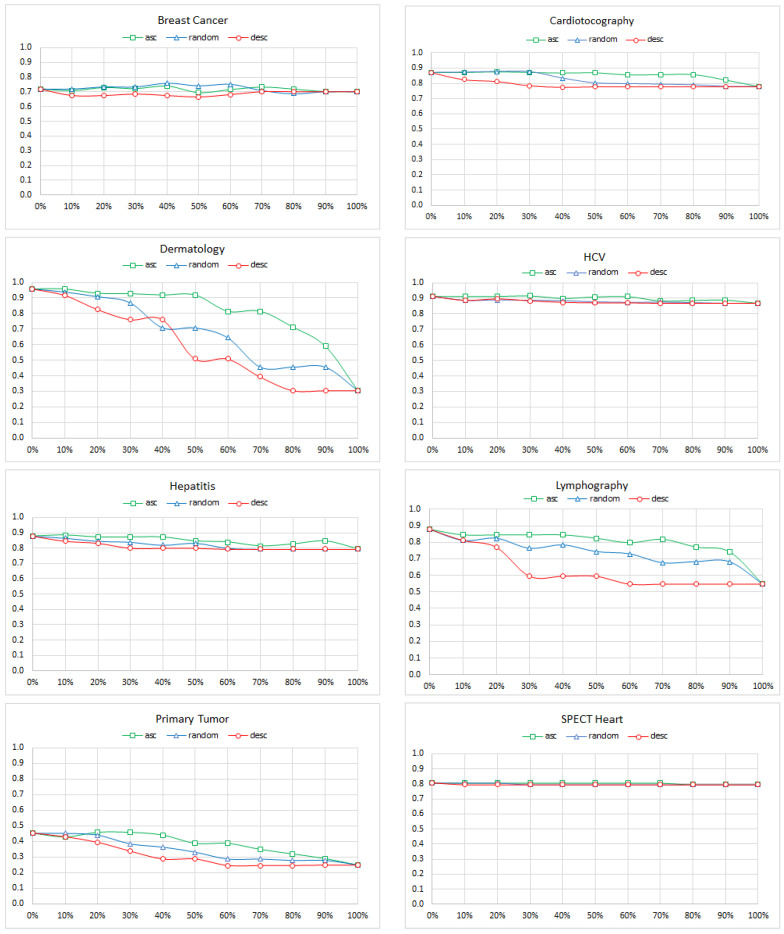
The AUC of the Breast Cancer, Cardiotocography, Dermatology, HCV, Hepatitis, Lymphography, Primary Tumor, and SPECT Heart models as a function of the percentage of edges removed. The colors indicate the ascending, random, and descending order of the strengths of the removed edges.

We started by calculating each edge of a gold standard BN model from Table 2 its strength. Arc strength is a static measure that does not change with the structural changes of the model. Subsequently, we proceeded with removing the edges one by one in one of the three orders: (a) from the weakest to strongest (ascending order), (b) the random order, and (c) from the strongest to weakest (descending order). We calculated the AUC measure for the most prevalent class (bold in Table 3) after each removal. Figure 5 shows the results. We can see that the edge removal initially had a smaller effect on the accuracy than node removal. Except for the Primary Tumor model, removing 10% or so edges has minimal effect on the accuracy. Yet, when the number of edges removed reached 30–40%, the deterioration in accuracy was evident.

### 2.6. Edge Reversal

**Figure 6:**
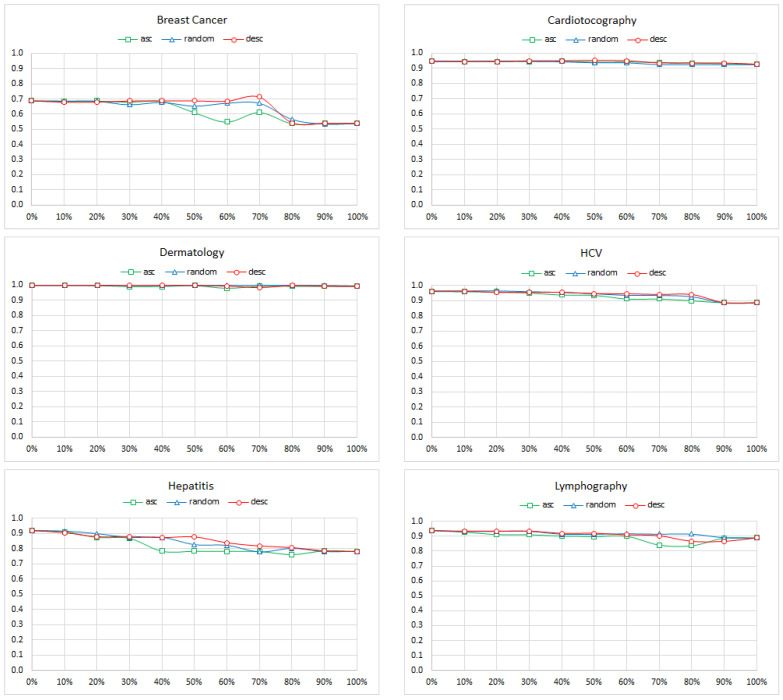

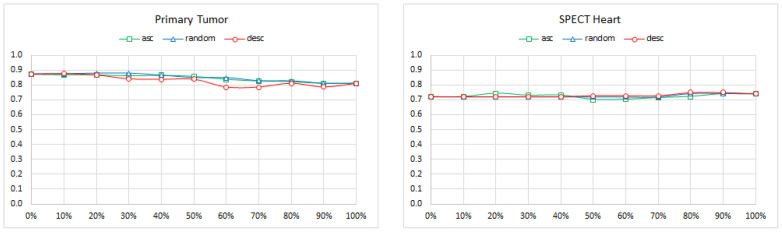
The AUC of the Breast Cancer, Cardiotocography, Dermatology, HCV, Hepatitis, Lymphography, Primary Tumor, and SPECT Heart models as a function of the percentage of edges reversed. The colors indicate the ascending, random, and descending order of the strengths of the reversed edges.

Similar to the previous experiment (edge removal), we started by calculating for each edge of a gold standard BN model from Table 2 its strength. Subsequently, we proceeded with reversing the direction of the edges one by one in one of the three orders: (a) from the weakest to strongest (ascending order), (b) random, and (c) from the strongest to weakest (descending order). However, we were forced to slightly deviate from the order. Since BNs are directed acyclic graphs and some reversals could lead to directed cycles in the graph, we were sometimes unable to reverse a specific edge. In such cases, we postponed the reversal of the edge in question, trying the next edge in the order until we encountered an edge that could be reversed. The omitted edges always remained at the beginning of the queue and were reversed as soon as it was possible. It is fairly easy to prove that this procedure only terminates after all the edges have been reversed. We calculated the AUC measure for the most prevalent class (bold in Table 3) after each reversal. Figure 6 shows the results. Here, the results are different from the first two experiments. It looked like the effect of the edge reversal, even though it affected the overall accuracy of the model, was minimal, even if most of the edges were reversed. In case of the Breast Cancer model, the accuracy deterioration was larger and started around 70% of the reversed edges. It was almost certainly due to the effect of the ABN structure. When all the arcs had been reversed, the conditional probability table (CPT) of the class node (the *recurrence* node in Figure 1, right) contained 299,376 probability distributions. Reliably learning these from the 286 data records of the *Breast Cancer* data set is impossible and has to take a toll in the model quality.

## 3. Discussion

The main result from our analysis is that structural errors lead to a rather fast deterioration in model quality, as measured by the model’s ability to discern its most likely class from other classes. This confirms the popular belief in the BN community that model structure is important, more important than its parameters. Still, the BN models that we studied seemed to be forgiving toward single errors. For most, although not all, models as many as 10% of nodes missing, 20% of edges missing, and 70% of wrongly oriented edges, leads to minimal loss of models accuracy Omitted important nodes was the most critical deficiency. This was followed by omitted important edges. Having a wrong edge orientation seemed the most forgiving. While each of these are a serious structural error, the possible presence of multiple paths or some missing independencies through wrong edge orientations have a likely smaller effect on the joint probability over all nodes.

In our experiments, the parameters of the mutilated models were re-learned from data records, which meant that the joint probability distribution modeled by the BN did not depart too far from the original, ideal joint probability distribution. It became important to look deeper into the model’s ability to discern various classes. The overall accuracy, which worked well when analyzing the impact of noise on the model quality, was too dependent on the *a priori* probability distributions over various classes (prevalence). We propose using the AUC measure as a good indication of the deteriorating quality of a model.

One weakness of our study is that we relied on structure learning algorithms to derive our initial gold standard models. One might think that using models that have their structures elicited from experts would be more realistic. On the other hand, any possible error introduced by the expert could be accidentally repaired by the mutilation, and the accuracy of the original model would not be the best possible. Also, a practical obstacle is that real models with associated real data sets are close to impossible to find.

An anonymous reviewer for Entropy suggested using different starting points in our experiments, i.e., different gold standard networks, which are learned by various structure learning algorithms. We believe that this would enhance our analysis only if these algorithms would result in significantly different structures. Partial structure learning algorithms (Naive Bayes, ANB, and Tree Augmented Naive Bayes) do not learn the causal structure of the domain, and their output is unlikely to be close to the models produced by human experts. The PC algorithm, a prominent causal structure learning algorithm, is known to produce dense structures for small data sets (this is the case in seven out of the eight selected data sets). This has to do with independence tests–null hypothesis, which is hard to reject when the number of data records is small. The Greedy Thick Thinning algorithm is based on BSA and produces graphs that are similar. Still, we conducted an experiment in which we started with gold standard structures originating from each of the six available structure learning algorithms implemented in GeNIe, but we obtained no additional insights that are worth reporting.

Another possible direction of further studies is looking deeper at the structural properties, for example, on the structural changes to the Markov blanket [3] of class nodes. In a way, cross-entropy captures some of these structural properties, as nodes that are the closest have the largest influence on the class node [21].

While our result is merely a single data point that sheds light on the hypothesis in question, we can confirm that Bayesian networks are sensitive to errors in their structures. This being said, we have observed that a handful of errors, especially those among less important variables, does not impact the overall accuracy too much. We caution that further empirical studies on this topic should be based on appropriate accuracy measures that look deep enough into the model’s ability to distinguish between various diseases.

## Figures and Tables

**Table 1:** The medical data sets selected for our experiments.

Data Set	Citation	Instances	Variables	Variable Types	Classes	Missing Values
*Breast Cancer*	[12]	286	10	Categorical	2	0.31%
*Cardiotocography*	[13]	2126	22	Categorical, real	3	—
*Dermatology*	[14]	366	35	Categorical, real	6	0.06%
*HCV*	[15]	615	13	Categorical, real	5	0.2%
*Hepatitis*	[16]	155	20	Categorical, real	2	5.4%
*Lymphography*	[17]	148	19	Categorical, integer	4	—
*Primary Tumor*	[17]	339	18	Categorical, integer	20	3.7%
*SPECT Heart*	[18]	267	23	Categorical	2	—

**Table 2:** The basic properties of the BN models used in our experiments.

Model	Number of Nodes	Average Number of States	Mean in-Degree	Number of Edges	Number of Parameters
Breast Cancer	10	4.50	1.40	14	200
Cardiotocography	22	2.91	2.86	63	13,347
Dermatology	35	3.94	0.83	29	2032
HCV	13	3.15	1.38	18	312
Hepatitis	20	2.50	1.90	38	465
Lymphography	19	3.00	1.05	20	300
Primary Tumor	18	3.17	1.83	33	877
SPECT Heart	23	2.00	2.26	52	290

**Table 3:** The number of classes and their distribution in the Bayesian network models used in our experiments.

Model	Number of Classes	Prevalence of Various Classes in the Data Set
Breast Cancer	2	(**70.3%**, 29.7%)
Cardiotocography	3	(**77.8%**, 13.9%, 8.3%)
Dermatology	6	(**30.6%**, 19.7%, 16.7%, 14.2%, 13.4%, 5.5%)
HCV	5	(**86.7%**, 4.9%, 3.9%, 3.4%, 1.1%)
Hepatitis	2	(**79.2%**, 20.8%)
Lymphography	4	(**54.5%**, 41.1%, 2.9%, 1.5%)
Primary Tumor	20	(**24.8%**, 11.5%, 8.6%, 8.3%, 7.1%, 7.1%, 5.9%, 4.7%, 4.1%, 4.1%, 2.9%, 2.7%, 2.4%, 2.1%, 1.8%, 0.6%, 0.6%, 0.3%, 0.3%, 0.3%)
SPECT Heart	2	(**79.4%**, 20.6%)

## Data Availability

All of the data sets described in this paper are openly available in the University of California Irvine Machine Learning Repository at https://archive.ics.uci.edu/ (accessed on 1 October 2024).

## Abbreviations

The following abbreviations are used in this manuscript:

ACC	Diagnostic accuracy (the proportion of correct diagnoses and a measure of a model’s quality)
ANB	Augmented Naive Bayes (a structure learning algorithm)
AI	Artificial Intelligence
API	Application Programming Interface
AUC	Area Under the (ROC) Curve (a measure of model’s ability to detect a single class)
BN	Bayesian Network
BSA	Bayesian Search (a structure learning algorithm)
CPT	Conditional Probability Table
VOI	Value Of Information (a measure of the worth/importance of a potential observation variable)
